# Estimating dose-response for time to remission with instrumental variable adjustment: the obscuring effects of drug titration in Genome Based Therapeutic Drugs for Depression Trial (GENDEP): clinical trial data

**DOI:** 10.1186/s13063-019-3810-9

**Published:** 2020-01-03

**Authors:** Jennifer Hellier, Richard Emsley, Andrew Pickles

**Affiliations:** Biostatistics and Health Informatics Department, Institute of Psychiatry, Psychology & Neuroscience, King’s College London, De Crespigny Park, London, SE5 8AF UK

**Keywords:** Depression, Dose response, Instrumental variables, Survival analysis, Threshold regression, Time to remission

## Abstract

**Background:**

Threshold regression, in which time to remission is modelled as a stochastic drift towards a boundary, is an alternative to the proportional hazards survival model and has a clear conceptual mechanism for examining the effects of drug dose. However, for both threshold regression and proportional hazard models, when dose titration occurs during treatment, the estimated causal effect of dose can be biased by confounding. An instrumental variable analysis can be used to minimise such bias.

**Method:**

Weekly antidepressant dose was measured in 380 men and women with major depression treated with escitalopram or nortriptyline for 12 weeks as part of the Genome Based Therapeutic Drugs for Depression (GENDEP) study. The averaged dose relative to maximum prescribing dose was calculated from the 12 trial weeks and tested for association with time to depression remission. We combined the instrumental variable approach, utilising randomised treatment as an instrument, with threshold regression and proportional hazard survival models.

**Results:**

The threshold model was constructed with two linear predictors. In the naïve models, averaged daily dose was not associated with reduced time to remission. By contrast, the instrumental variable analyses showed a clear and significant relationship between increased dose and faster time to remission, threshold regression (velocity estimate: 0.878, 95% confidence interval [CI]: 0.152–1.603) and proportional hazards (log hazards ratio: 3.012, 95% CI: 0.086–5.938).

**Conclusions:**

We demonstrate, using the GENDEP trial, the benefits of these analyses to estimate causal parameters rather than those that estimate associations. The results for the trial dataset show the link between antidepressant dose and time to depression remission. The threshold regression model more clearly distinguishes the factors associated with initial severity from those influencing treatment effect. Additionally, applying the instrumental variable estimator provides a more plausible causal estimate of drug dose on treatment effect. This validity of these results is subject to meeting the assumptions of instrumental variable analyses.

**Trial registration:**

EudraCT, 2004–001723-38; ISRCTN, 03693000. Registered on 27 September 2007.

## Background

### Clinical perspective

When an exposure of interest is subject to unmeasured confounding, instrumental variable (IV) models can be used to test and estimate the causal effect of exposures on disease-related outcomes [1, 2]. IV estimators have been described and evaluated for use with data in a time-to-event setting [3]. We explore the practical application of these methods in the context of illustrative trial data, drawing on the virtues of trial design and randomised treatment as an IV [4]. This work is extended to show the benefits of utilising threshold regression with an IV estimator method, a novel approach for researchers who may wish to fit models that estimate causal effects in a survival setting. This manuscript describes a novel method development informed by application of trial data of antidepressant dose response.

### Dose response in time to remission

**Fig. 1:**
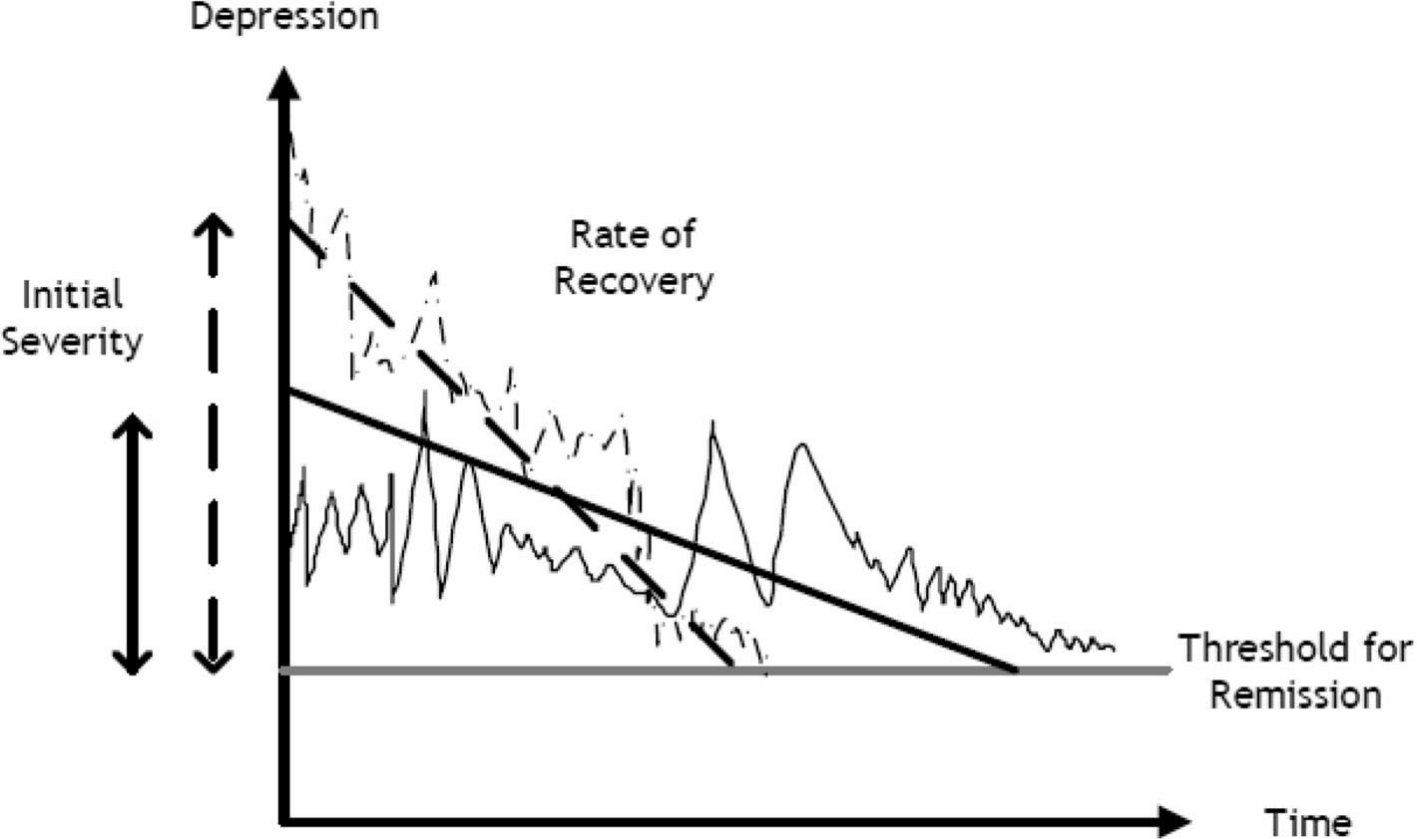
The inverse Gaussian (IG) model. Two hypothetical time paths of depression (one *solid* and one *dashed*) showing actual and linear smoothed depression scores. The participant’s depression score under the IG model evolves as Brownian motion with a trend but with random local changes

From both patient and clinical service perspectives, reducing time to remission is an important therapeutic goal. In the treatment of depression, many researchers have suggested that achieving remission should be viewed as the primary goal [5–7]. The most commonly used tool for the analysis of remission times is the Cox proportional hazards (PH) survival model, which estimates the effect of therapy on the relative risk of remission. While statistically elegant, and often giving an efficient and parsimonious characterisation of the effects of therapy, this model is rather agnostic as to the underlying process of recovery. Models based on a more explicit conceptualisation of the process can sometimes be more helpful [8, 9], particularly where there are multiple ways in which a variable may have an effect. One such model is the inverse Gaussian (IG) survival model. In the IG model, a patient can be considered as starting some distance away from the threshold for remission and drifting with some velocity towards the threshold. Their progress is not assured, but is probabilistic, and some may drift the wrong way and never remit. Part of the appeal of the model is that it distinguishes patient characteristics that might be associated with initial severity, conceived of a distance from the threshold, from those characteristics associated with velocity of recovery. Figure 1 illustrates that for two patients, the time taken to remission is necessarily a function of both initial severity and rate of recovery.

Reaching the threshold for remission is determined by the patient’s initial severity or baseline position and the rate of recovery or progression towards or away from remission.

A common complication of time to remission studies is the necessity to titrate each patient’s drug dose over the first few weeks to a level considered both therapeutic and acceptable [10]. This practice can result in bias in the naïve estimates of drug dose-response, since it is common for the most responsive patients to be titrated to a lower dose than those with more refractive symptoms, for whom dose is often progressively increased in the hope of achieving an improvement in symptoms. This practice results in routine analysis suggesting higher dose being associated with longer time to remission, something which we know to be a pharmacologically implausible. A possible solution to this is to estimate the effect of dose using an IV approach [2, 11]. This requires having a variable (the instrument) that influences dose but does not influence time to remission except through its influence on dose, and such a variable can be obtained by exploiting the random treatment assignment of a randomised controlled trial (RCT).

As a member of the class of so-called non-collapsible models, the routinely used Cox PH model does not lend itself to the IV approach [3], whereas the IG model does [12]. In this paper, we demonstrate for the first time that the combination of the IG model and IV estimation proves to be a straightforward way to recover dose response effects with less titration bias. It aims to contrast methods for combining an IV estimator with PH and threshold regression survival models. We illustrate these methods for the Genome-Based Therapeutic Drugs for Depression (GENDEP) trial that compared two types of anti-depressant in people with depression. Estimated causal effects can be biased by confounding and these methods can be used in practice to minimise such bias in a survival setting.

## Methods: illustrative trial data

### Trial design

We analysed data from the Medical Research Council-funded GENDEP study, a multicentre open-labelled, randomised clinical trial [13], compliant with CONSORT [14]. The study was carried out in nine European academic psychiatric centres and was designed to evaluate therapeutic response to two antidepressants. All participants provided a written consent after the procedures were explained. GENDEP is registered at EudraCT and ISRCTN.

### Participants

The GENDEP study included 811 adult men and women with a current diagnosis of major depressive disorder, established using the Clinical Assessment in Neuropsychiatry interview (SCAN version 2.1). Recruitment was restricted to people of white European ethnicity. People with (or had a family history) of bipolar disorder or schizophrenia and active substance dependence were excluded. Participants were also excluded if they had contraindications or a history of lack of efficacy or adverse reaction to both study medications. A total of 468 participants had no contraindications to either drug and were randomly allocated to receive escitalopram or nortriptyline. The present study includes 380 (81%) of these participants, where treatment dosing was recorded.

### Measures and covariates

Depression was measured weekly and here we focus on the primary outcome, the clinical rated 10-item Montgomery-Åsberg Depression Rating Scale (MADRS) [15]. Remission was calculated at each study week, defined as a MADRS measure of ≤ 10 for the current and remainder of available measures. Time to the week remission began was calculated from randomisation to the first week of remission, over a maximum of 12 weeks. Censored observations are those participants whom dropped out of or switched treatment.

All other covariates utilised in these analyses were measured at baseline before randomisation and the initiation of treatment. Body weight and height were measured by calibrated scales. Body mass index (BMI) was calculated as a continuous measure of body weight relative to height (kg/m^2^). Other baseline covariates considered were MADRS score, age (years) at randomisation, age (years) at time of depression onset, duration of current episode measured in weeks and gender.

### Intervention and dosing

Eligible participants were randomised to receive a 12-week treatment. The two randomised antidepressants represented the two most common mechanisms of action among commonly used antidepressants and have a good efficacy record. Escitalopram is highly selective inhibitor of the serotonin transporter [16]. Nortriptyline is a tricyclic antidepressant with an affinity for the noradrenaline transporter 100 times higher than that for the serotonin transporter [17].

Trial medication was started immediately after the first assessment. Escitalopram was initiated at 10 mg daily and increased to a target dose of 15 mg daily within the first two weeks (unless adverse effects limited dose increase) and could be increased to 20 mg daily (and up to 30 mg if there was clinical agreement that a higher dose was needed). With similar clinical guidelines: nortriptyline was initiated at 50 mg daily and titrated to a target of 100 mg within the first two weeks (unless adverse effects limited dose increase); this could be increased to 150 mg with a maximum of 200 mg (if there was clinical agreement that a higher dose was needed). Relative dose was determined weekly by division of the current dose by the British National Formulatory’s (https://www.bnf.org/) recommendation of maximum daily dose: 20 mg daily and 150 mg/day for escitalopram and nortriptyline, respectively. The parameter of interest was the average relative dose, for which the denominator was the number of weeks a measure of dose was available, of a possible 12 weeks.

All available weekly data in response to antidepressant treatment with dosing measures, MADRS scores and relevant covariates were included in the analyses.

## Methods: analysis models

### Kaplan–Meier

Time to remission is initially presented as Kaplan–Meier plots. Relative dose is dichotomised using the median to demonstrate survival functions by high and low dose.

### Inverse Gaussian model

Threshold regression refers to a statistical model for time to event data, in which the time to the event is defined as the first hitting of an absorbing boundary by an underlying stochastic process. We use the version of the IG model termed ‘Wiener Process with Absorption’ in the review by Aalen and Gjessing in 2001 [18] and previously applied in psychiatry by Crouchley and Pickles in 1991 [19]. In this model, the distribution of time to remission (*t*) is given by:

$$ {\boldsymbol{f}}_{\boldsymbol{i}\boldsymbol{g}}\left(\boldsymbol{t}\right)=\left({\left({\boldsymbol{c}}_{\boldsymbol{i}}/{\boldsymbol{\sigma}}_{\boldsymbol{i}}\right)\left(\mathbf{2}\boldsymbol{\pi } {\boldsymbol{t}}^{\mathbf{3}}\right)}^{-\mathbf{1}/\mathbf{2}}\right)\mathbf{\exp}\left(-{\left({\boldsymbol{c}}_{\boldsymbol{i}}-{\boldsymbol{\mu}}_{\boldsymbol{i}}\boldsymbol{t}\right)}^{\mathbf{2}}/\left(\mathbf{2}{\boldsymbol{\sigma}}_{\boldsymbol{i}}^{\mathbf{2}}\boldsymbol{t}\right)\right) $$

The IG distribution depends on the mean and variance parameters of the underlying Wiener process (*μ* and *σ*) and the initial patient status (*c*), where *c*_*i*_/*σ*_*i*_ is the patient’s initial distance from the threshold, *μ*_*i*_/*σ*_*i*_ is the velocity of the patient towards or away from the threshold. In Fig. 1, the patients’ initial distance (*c*_*i*_/*σ*_*i*_) is shown as their baseline depression severity; the velocity towards the threshold (*μ*_*i*_/*σ*_*i*_) is a measure that determines their rate of recovery.

Most explanatory variables (denoted *x*_1_, *x*_2_, …, *x*_*m*_) could be linked to both initial severity (distance from the remission threshold) and the rate of symptom improvement (velocity). However, randomised treatment or post-randomisation variables such as dose (denoted *z*_1_, *z*_2_, …, *z*_*m*_), cannot be associated with initial severity, but only to the rate of symptom improvement. Initial distance is linked to baseline covariates with an exponential function:

$$ {\boldsymbol{c}}_{\boldsymbol{i}}/{\boldsymbol{\sigma}}_{\boldsymbol{i}}=\boldsymbol{\exp}\left({\boldsymbol{\alpha}}^{\prime }{\boldsymbol{x}}_{\boldsymbol{i}}\right)=\boldsymbol{\exp}\left({\boldsymbol{\upalpha}}_{\mathbf{1}}{\mathbf{x}}_{\mathbf{1}}+{\boldsymbol{\upalpha}}_{\mathbf{2}}\ {\mathbf{x}}_{\mathbf{2}}+\dots +{\boldsymbol{\upalpha}}_{\mathbf{m}}{\mathbf{x}}_{\mathbf{m}}\right)=\boldsymbol{\exp}\left({\boldsymbol{\theta}}_{\mathbf{1}}\right) $$

whereas the velocity is linked to covariates as a linear function, given the matrix *γ* = (*x*_1_, *x*_2_, …, *x*_*m*_, *z*_1_, *z*_2_, …, *z*_*m*_):

$$ {\boldsymbol{\mu}}_{\boldsymbol{i}}/{\boldsymbol{\sigma}}_{\boldsymbol{i}}=\left({\boldsymbol{\beta}}^{\prime }{\boldsymbol{\gamma}}_{\boldsymbol{i}}\right)=\left({\boldsymbol{\upbeta}}_{\mathbf{1}}{\boldsymbol{\gamma}}_{\mathbf{1}}+{\boldsymbol{\upbeta}}_{\mathbf{2}}\ {\boldsymbol{\upgamma}}_{\mathbf{2}}+\dots +{\boldsymbol{\upbeta}}_{\mathbf{m}}{\boldsymbol{\upgamma}}_{\mathbf{m}}\right)={\boldsymbol{\theta}}_{\mathbf{2}} $$

where {*α*}and {*β*} are coefficients to be estimated. The simple linear function implies that the predicted direction of drift can be both towards and away from the boundary and that remission is not inevitable even in the long run. Unlike the PH model, coefficient estimates from the linear predictor for velocity do not systematically vary with the inclusion of other uncorrelated variables.

The associated survival function:

$$ \boldsymbol{S}\left(\boldsymbol{t}\right)=\boldsymbol{\phi} \left(\frac{\boldsymbol{c}-\boldsymbol{\mu} \boldsymbol{t}}{\boldsymbol{\sigma} \boldsymbol{\surd}\boldsymbol{t}}\right)-\boldsymbol{\exp}\left(\frac{\mathbf{2}\boldsymbol{c}\boldsymbol{\mu}}{{\boldsymbol{\sigma}}^{\mathbf{2}}}\right)\boldsymbol{\phi} \left(\frac{-\boldsymbol{c}-\boldsymbol{\mu} \boldsymbol{t}}{\boldsymbol{\sigma} \boldsymbol{\surd}\boldsymbol{t}}\right) $$

where *ϕ*(.) is the cumulative standard normal distribution. The hazard rate can be calculated, as usual, from *h*(*t*) = *f*(*t*)/*S*(*t*). Given there are three parameters in the IG distribution, *c*, *μ* and *σ*, but the distribution only depends on these through the functions of *c*/*μ* and *μ*/*σ*, from a statistical point of view, there are two free parameters.

It is important to note, in a randomised trial, the IG model could be used to estimate the intention-to-treat analysis, where treatment allocation could feature alone in the velocity linear predictor. However, in our analyses the estimand of interest is dose response and dose is measured after randomisation and therefore could be subject to confounding. One method for dealing with this is to use an IV approach to get unbiased estimates of dose. This is the first time IG models have been used with IV estimation to estimate dose response.

Covariates may have a different impact on the initial distance or velocity. The two sets of covariates used in the velocity or distance linear predictors can be partially or entirely different. Post-randomisation variation in variables were only included in the velocity linear predictor [18]. In this application, different variables were tested as potential predictors for *exp*(*θ*_1_) and *θ*_2_. Baseline MADRS score, duration of current depressive episode and gender (reference category male) were considered possible predictors of distance from remission. Age of onset, BMI and relative dose were used in the second (velocity) linear predictor. The IG model was fitted by maximum likelihood using a purpose written program in *Stata* 14, *Stata* code is given in Additional file 1.

### Cox model

Cox PH regression is a well-known model for analysing remission times [20]. For our purposes, we note that the effect of predictors of time to remission enter the model multiplicatively on the rate of remission by exponentiation of a regression type linear predictor:

$$ {\boldsymbol{\uplambda}}_{\mathbf{i}}\left(\mathbf{t}\right)={\boldsymbol{\uplambda}}_{\mathbf{0}}\left(\mathbf{t}\right)\mathbf{\exp}\left({\boldsymbol{\upbeta}}_{\mathbf{1}}{\mathbf{x}}_{\mathbf{1}}+{\boldsymbol{\upbeta}}_{\mathbf{2}}\ {\mathbf{x}}_{\mathbf{2}}+\dots +{\boldsymbol{\upbeta}}_{\mathbf{m}}{\mathbf{x}}_{\mathbf{m}}\right) $$

where *λ*_*i*_(*t*) is the hazard function at time *t*, here *i* is a subscript for observation and the *xs* are the covariates with effects estimated by their corresponding coefficients {β}. The constant *λ*_0_(*t*) represents the baseline hazard function.

The Cox model, though it has many strengths, is non-collapsible, since the marginal regression parameter for a covariate is not equal to the conditional parameter when other covariates are included in the model [21, 22] . This implies that the magnitude of an estimated coefficient depends upon the inclusion or exclusion of another predictor variable, even when these two variables are uncorrelated.

We compared estimates from the IG model with the Cox PH model. We included baseline MADRS score, duration of depression, gender, age of depression onset, baseline BMI and average relative dose. PH were assumed. Estimates are presented as log hazard ratios (HR) with associated 95% confidence intervals (CI).

### Instrumental variable estimation using the two-stage residual inclusion approach

A correct IV analysis rests on three fundamental assumptions [4, 23]: (1) the instrument must be correlated with the exposure of interest (relevance); (2) the instrument affects the outcome only through its relation to the exposure of interest, there is no residual direct effect of treatment on the outcome (exclusion restriction); and (3) the instrument must be independent of the confounder (exogeneity).

Randomised antidepressant treatment was used as an IV using the two-stage residual inclusion (TSRI) approach [3]. The first stage of this method involved estimating the exposure of interest average relative dose, *y*_*D*_, from our IV variable, randomised treatment. This regression model was further adjusted for the baseline covariates and age at randomisation, covariate set {C}. Inclusion of other measured covariates in this step can help improve precision of the IV estimator:

$$ {\boldsymbol{y}}_{\boldsymbol{D}}={\boldsymbol{c}}_{\mathbf{0}}+{\boldsymbol{c}}_{\boldsymbol{z}}\boldsymbol{Z}+{\boldsymbol{c}}_{\boldsymbol{c}}\boldsymbol{C}+\boldsymbol{\varDelta} $$

The residuals from the first-stage regression, Δ, can be considered as estimating the effects of other uncontrolled factors that influence a patient’s dose. In the second stage of this method, we estimate the survival model, but we include among the predictor variables the residuals from the first stage together with the patient’s relative dose, *D*:

$$ \overline{\boldsymbol{h}}\left(\boldsymbol{t}|\boldsymbol{D},\boldsymbol{Z}\right)={\overline{\boldsymbol{b}}}_{\mathbf{0}\left(\boldsymbol{t}\right)}+{\boldsymbol{b}}_{\boldsymbol{d}}\left(\boldsymbol{t}\right)\boldsymbol{D}+{\boldsymbol{b}}_{\boldsymbol{c}}\boldsymbol{C}+{\boldsymbol{\rho}}_{\mathbf{0}}\left(\boldsymbol{t}\right)\boldsymbol{\Delta} $$

where $\overline{\boldsymbol{h}}\left(\boldsymbol{t}|\boldsymbol{D},\boldsymbol{Z}\right)$ denotes the observed hazard function of T given (***D***, ***Z***) evaluated at time time, *t*.

The second stage was implemented in both survival models. Confidence intervals and *p* values were based on 1000 non-parametric bootstrap samples to account for the two-stage approach.

## Results

Of the participants for whom antidepressant dose data were available, 196 were allocated to escitalopram and 184 to nortriptyline. Of these, 306 (80.3%) completed at least eight weeks of treatment. Completion rates were higher for escitalopram, 134 in the escitalopram group and 99 in the nortriptyline group had outcome data available for week 12. Additional file 2 details the baseline characteristics of participants included in the analyses. The trial population was mainly women with a mean age of 42 years (SD = 11); just over half the participants were married or cohabiting. For the majority, depressive onset was 10 years before the beginning of the study and most had had two previous depressive episodes. The current episode was approximately 20 weeks in duration (SD = 17). Half of the participants had taken antidepressants previously. BMI indicated average weight and baseline MADRS scores were high (mean = 30, SD =6).

At week 8, the median dose of escitalopram was 15 mg (interquartile range 10–20 mg) and the median dose of nortriptyline was 100 mg (interquartile range 75–125 mg). Overall average relative dose was higher for escitalopram 0.74 than nortriptyline 0.61. In the total sample, there was a weak *positive* correlation of the average relative dose with the final week 12 MADRS score (r = 0.0726, *p* = 0.27) and a significant *positive* correlation with time to remission (r = 0.2668, *p* < 0.01), reflecting that higher doses may be prescribed for those patients not responding well to treatment. Furthermore, of those patients given an escalating dosing regimen at some period during the 12 weeks of study, the majority did not reach remission: 61% (n = 78/128) and 71% (104/145) for escitalopram and nortriptyline, respectively.

**Fig. 2:**
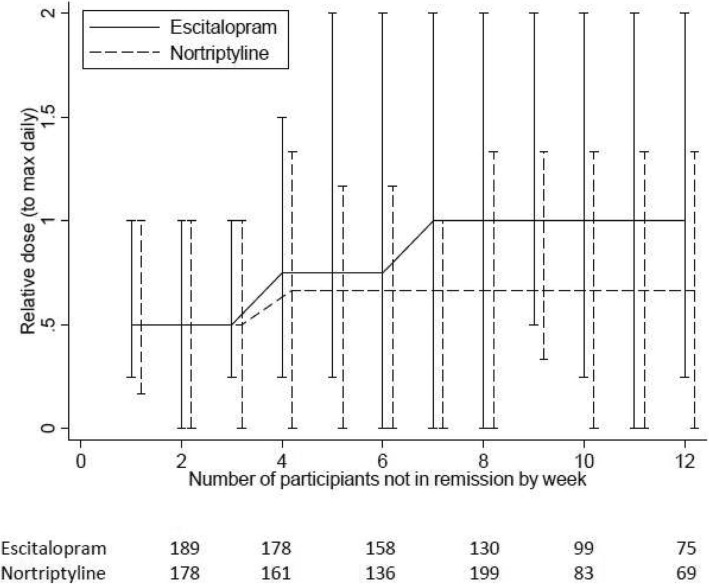
Median relative dose. *Plots* are shown for escitalopram and nortriptyline by trial week for those participants not in remission. *Error bars* are minimum and maximum quantities

Figure 2 shows, over the 12 study weeks, the relative dose for those participants not in remission. The increasing dosing regimens are apparent for both treatments. For those not in remission, the median relative dose for escitalopram was 100% of the prescribing guidelines from week 7 onwards and the maximum dose reaches twice that of the recommended daily dose. We know from previous reports of this trial that the more highly dosed escitalopram regimen proved to be more efficacious than the lower-dosed nortriptyline [13].

### Regression analysis

Table 1 showed that relative dose was strongly predicted by randomised treatment, with an F-statistic of 32 [24] and beta = − 0.131 (95% CI − 0.18 to − 0.09), implying that the relative daily dose of nortriptyline on average over the 12-week period was 13% lower than escitalopram. Sex and age showed marginally significant associations, but, perhaps surprisingly, prior duration and age of onset of depression and BMI were unrelated to relative dose. We extracted the residuals from this regression for inclusion in subsequent survival analyses. Since treatment allocation was random, these residuals meet the assumptions required for a TSRI estimator. We refer to these as Stage 1 residuals.

**Table 1:** Predicting relative dose using linear regression. Regression output for prediction of relative dose from treatment and baseline covariates. The association measure is the regression coefficient (SE), with 95% CIs

Relative dose	Coefficient (SE)	95% CI	*p* value
MADRS	0.003 (0.002)	− 0.001 to 0.006	0.117
Prior duration of depression (weeks)	0.000 (0.001)	− 0.001 to 0.001	0.853
Sex (female)	0.046 (0.024)	0.000 to 0.093	0.052
Age of depression onset (years)	0.001 (0.001)	−0.002 to 0.004	0.399
BMI (kg/m^2^)	0.003 (0.002)	−0.001 to 0.008	0.149
Treatment (nortriptyline)	−0.131 (0.023)	−0.175 to − 0.086	< 0.001
Age (years)	− 0.002 (0.001)	− 0.005 to 0.000	0.060

*BMI* body mass index, *CI* confidence interval, *MADRS* Montgomery-Åsberg Depression Rating Scale, *SE* standard error

**Fig. 3:**
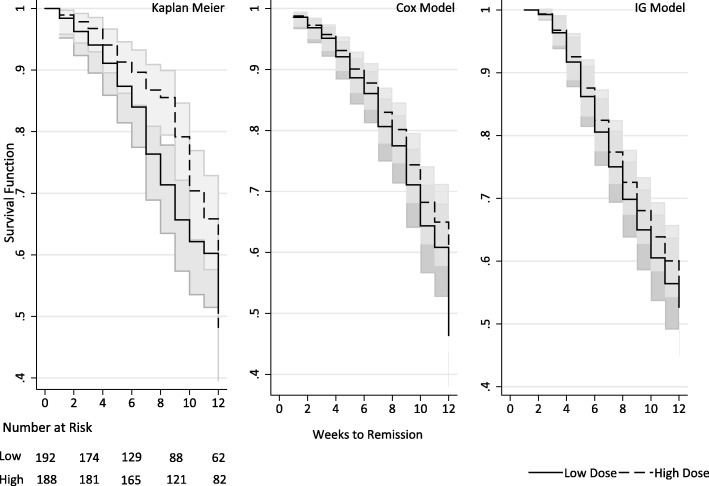
Time to depression remission. *Plots* show relative dose dichotomised by median split of average weekly dose. Analyses were adjusted for no further covariates. *Plots* from left to right: Kaplan–Meier; Cox model; IG model. *Shaded area* shows 95% pointwise CIs for Kaplan–Meier and Cox models, IG model 500 bootstrap replications of the survival function used to estimate a standard error and CI

Overall, the average time to remission was 7.96 weeks (95% CI = 7.4–8.5). In total, we observed 143 participants in remission over 3269 treatment weeks. In Fig. 3, survival functions from Kaplan–Meier, Cox and IG models are plotted for median dichotomised high and low relative doses and unadjusted for covariates. All three suggest that a higher dose is associated with increased time to remission in all three methods, mean time to remission was 7.82 weeks (95% CI = 7.3–8.3) for lower doses and 9.40 weeks (95% CI = 9.0–9.8) for higher doses. We suspected that these counterintuitive results were explained by titration bias.

Table 2 shows results from the Cox and IG models. For each covariate, two rows of estimates are shown: those in the first row are for the standard analysis while those in the second row were obtained using the TSRI estimator from models that also included the Stage 1 residuals as an additional covariate. In the standard Cox model, while, as expected, baseline MADRS score was highly significant, with log HR = − 0.051 (95% CI = − 0.079 to − 0.023) implying higher severity being associated with longer time to remission, higher relative dose was not significantly associated and the estimated coefficient also implied a decreasing log HR = − 0.180 (95% CI = − 0.933 to 0.573). Like the simple summary statistics suggested, this implies an increase in relative dose was associated with a decreased chance of remission and longer time to remission.

**Table 2:** Observational analysis of relative dose. Regression output for dose response on time to remission under the Cox and inverse Gaussian model with and without the IV stage 1 residuals introduced as a control variable

	Cox PH model						Inverse Gaussian model					
Time to remission	Log HR	95% CI	*p* value	With stage 1 residuals			CoEff	95% CI	*p* value	With stage 1 residuals		
				Log HR	95% CI	*p* value				CoEff	95% CI	*p* value
							Distance linear predictor					
MADRS	− 0.051	− 0.079 to − 0.023	< 0.001	− 0.056	− 0.088 to − 0.024	0.001	0.026	0.018 to 0.035	< 0.001	0.028	0.014 to 0.041	< 0.001
Duration of depressive episode (weeks)	− 0.009	− 0.020 to 0.001	0.086	− 0.010	− 0.020 to 0.001	0.061	0.006	0.002 to 0.009	0.001	0.006	0.002 to 0.010	0.003
Sex (female)	− 0.153	− 0.492 to 0.187	0.378	− 0.338	− 0.747 to 0.072	0.106	0.196	0.066 to 0.325	0.003	0.240	0.063 to 0.417	0.008
							Velocity linear predictor					
Age of onset (years)	− 0.007	− 0.024 to 0.009	0.378	− 0.007	− 0.024 to 0.011	0.453	− 0.002	− 0.006 to 0.003	0.457	− 0.002	− 0.00 to 0.003	0.522
BMI (kg/m^2^)	− 0.034	− 0.068 to − 0.001	0.050	− 0.039	− 0.076 to − 0.003	0.036	− 0.010	− 0.019 to − 0.002	0.020	− 0.012	− 0.021 to − 0.002	0.015
Relative dose	− 0.180	− 0.933 to 0.573	0.639	3.012	0.086 to 5.938	0.044	− 0.029	− 0.218 to 0.160	0.767	0.878	0.152 to 1.603	0.018
Stage 1 residuals				− 3.508	− 6.569 to − 0.447	0.025				− 1.011	− 1.774 to − 0.247	0.009

*BMI* body mass index, *HR* hazard ratio, *MADRS* Montgomery-Åsberg Depression Rating Scale, *PH* proportional hazard

The same pattern of response was found in the standard IG model. For the distance linear predictor, representing the logarithm of initial depression status, it is seen that baseline MADRS score is significant, with *p* < 0.001, with a positive regression coefficient signifying the initial distance (or baseline depression status from a remission boundary) tends to be higher for participants with higher baseline depression scores. The coefficient for duration of the depressive episode and gender are also positive, indicating that initial depression status was further from remission for women and those with a longer duration of depression. For the velocity linear predictor, the regression coefficients are all negative, indicating a negative effect slowing remission. Relative dose has a non-significant negative coefficient − 0.029 (95% CI = − 0.218 to 0.160), consistent with the unadjusted analyses seen in Fig. 3 and the standard Cox model in this table, where a higher relative dose delays remission.

For the IG model, when the Stage 1 residuals are included as an additional covariate in the velocity linear predictor, the coefficient of relative dose becomes highly significant (*p* = 0.018) and positive, showing a faster rate of recovery with increasing dose (beta = 0.878, 95% CI = 0.152–1.603), which makes theoretical sense. The estimates for the other covariates generally remain unchanged.

**Fig. 4:**
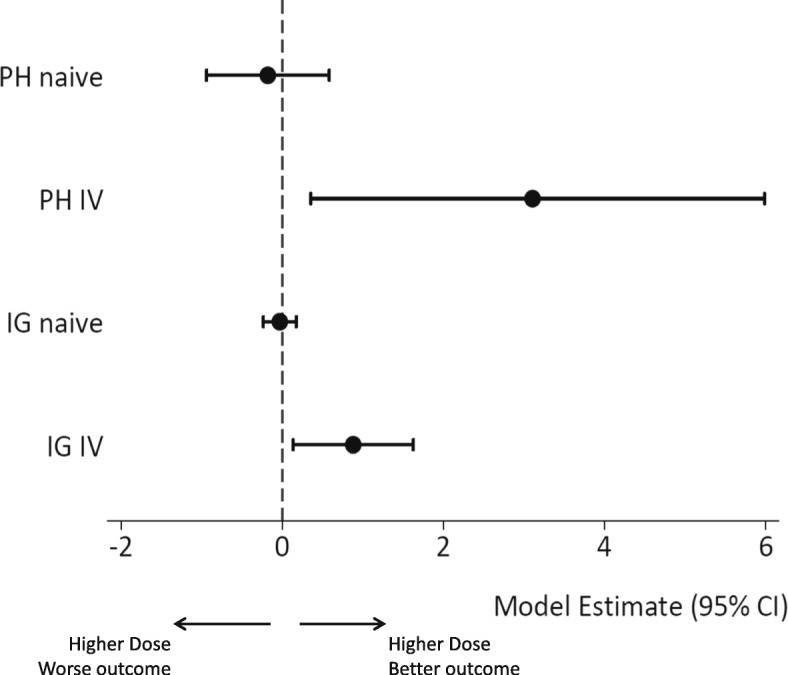
Results comparing naïve and IV model estimates from IG and Cox models. Regression estimates and 95% CI for dose effect on time to remission under the Cox (PH), log HR, and inverse Gaussian (IG), velocity coefficient, models. Estimates shown without (naïve) and with (IV) the stage 1 residuals introduced as control variables. Results from fully adjusted models

For the Cox model, the addition of the Stage 1 residuals as a covariate shows a similar effect in terms of the direction and significance, but there is clear evidence of the problems of non-collapsible in modelling relative risk, with the log HR estimate of 3.012 (95% CI = 0.086–5.938) implying an extreme HR of > 20. The results of the model comparisons are presented in Fig. 4.

In both models, we obtained further evidence of titration bias from the highly significant estimated effects of the first-stage residuals, such that high residuals strongly predicted longer time to remission (IG beta = − 1.101, 95% CI − 1.774 to − 0.247; Cox beta = − 3.508, 95% CI = − 6.569 to − 0.447). Hausman [25] showed that the test of the coefficient of the first-stage residuals is a test for the presence of unmeasured confounding. By including these residuals in the second stage, we correct for some of the confounding, known as endogeneity [26]. We can hypothesise that the residuals, the prescribing of higher (or lower) dose than expected given the patients’ characteristics and drug assignment, are representing a group of unmeasured variables that imply a poor (good) prognosis and a slow (fast) rate of recovery.

The results for all other covariates are essentially identical to the more naïve analyses as expected. The correlations of the residuals with these baseline covariates were all small.

## Discussion

In this paper, we have used a novel application of IV techniques in an IG model to estimate an unbiased relative dose response on time to remission relationship in the GENDEP study.

We demonstrate the benefits of these analyses estimating causal parameters accounting for confounding, rather than those that estimate associations. This is achieved through using a model with mechanistic underpinnings, together with a TSRI estimator to account for the unmeasured confounding that arose from the process of individual patient dose titration. The results for the trial dataset illustrate emphatically how the method can recover scientifically meaningful estimates of the dose–response relationship even where standard analysis gives estimates in the wrong direction.

Application of a TSRI method is intended to give an unbiased estimate of dose response on time to remission. The IV analyses showed a clear and significant relationship between increased dose and faster time to remission. Both the direction and magnitude of the dose response were changed by use of the IV approach, and though evident in both the IG and Cox models, the non-collapsibility of the Cox model made this model hard to interpret.

The methods proposed here demonstrate an important application of IV methodology that is clinically useful. We have shown how IV assumptions have led to the correction of an antidepressant titration bias in a randomised controlled trial. This application is especially important because treatment assignment can provide a perfect IV for confounding control and IV methods provide an alternative causal analysis.

We have seen that in spite of their different mathematical structures, the IG model and Cox regression give qualitatively similar findings. Recognising the strengths of the Cox regression and where its assumptions are valid, it should be used. There are, however, clear advantages in this setting for the IG model where, compared to a multiplicative model, the linear additive form of the equation predicting velocity is more easily interpreted in the IV setting [27], a property demonstrated in our results. Also, in having two linear predictors and thus two sets of coefficients for covariate effects, namely those associated with the distance and velocity, the IG model allows researchers to consider the mechanism of time to remission separately from the covariates that influence the initial depth of depression from those which influence the course of the depression progression after initiation of treatment. Additionally, the IG focuses on survival (remission), which may be of greater interest than HRs [28, 29]. The structure of the IG model is such that different choices in the threshold might not be expected to give rise to systematic variation in the estimated coefficients of the model, which would be an appealing property, this requires further investigation.

Tchetgen Tchetgen et al. [3] introduce the TSRI and a two-stage least squares regression based on using the predicted values from the first stage in place of the exposure variable as methods for IV estimation in the survival context. Although the two approaches are equivalent in their estimation [30], in this application we have chosen the TSRI approach. This approach can be extended to a binary exposure and we favour the explicit estimation of the coefficient for the residuals.

We have shown the implementation of an IV—randomised treatment—to estimate the average casual effect of an exposure—dose—on time to remission. The methods demonstrate that IV in survival context is easy to understand and apply once an appropriate IV has been identified. The use of this causal methodology rests on the validity of the assumptions for the IV [31] and the conditions that must be satisfied to achieve consistent estimates of the causal effects. The three main conditions that define an IV are: (1) treatment has a causal effect on dose; (2) treatment affects the time to remission only through the relative dose; and (3) there is no confounding of the effect of treatment on time to remission. We can argue that randomised treatment satisfies conditions (1) and (3). Furthermore, the first stage of the IV method demonstrates that allocation is strongly correlated with the exposure of interest, relative dose [1]. To explore the exclusion restriction in the context of our modelling, we tested an interaction between treatment and centre as an alternative IV which would then allow for a direct effect of treatment in the second stage model. Treatment allocation was not associated with time to remission in the presence of the relative dose (Cox log HR = – 0.744, 95% CI = − 3.190 to 1.702, IG velocity estimate = − 0.015, 95% CI = − 0.308 to 0.278).

### Limitations of the study

One weakness in our study is the plausibility of the relative dose variable. We have used a flexible escalating dose trial, with the two antidepressants follow a different dosing scheme. To analyse the effects of dose response, drug doses needed to be modelled on the same scale. We chose a relative comparison to prescribing daily maximums, averaging these over weeks of treatment. To fully explore the effect of dose, it could be argued that the role of plasma levels of antidepressants and their proximal metabolites should be modelled. However, plasma levels are not comparable between the two antidepressants and a standardised scale would still need to be derived that could be used for plasma metabolites of both drugs. Furthermore, we are making the assumption that the standardised dose effects for the two antidepressants are equivalent in our modelling and the mechanism of increased dose effect comparable. By averaging the relative dose over the 12 weeks, we are aware there may be some finer dosing effects where the rate of dose increase may affect the velocity to recovery that still requires further investigation and would be insightful future work.

Finally, these analyses were a subsample of the randomised trial population (n = 380, 81%); this may have biased our results if the subsample that had dosing information available is not representative of the whole trial population.

## Conclusions

In conclusion, we have shown that higher antidepressant dosing can predict quicker time to depression remission. We have further demonstrated the use of an innovative causal instrumental variable approach in a survival context. The results appear robust and meet the testable assumptions of the modelling. We have highlighted the benefit of an IG model for time-to-event analyses. Further study is needed to establish why some people do not respond well to higher doses of antidepressant treatment.

## Supplementary information

**Additional file 1:** Stata command routines. Table: GENDEP baseline demographic and clinical characteristics.

## Data Availability

Code for the IG model is available upon request to the first author, jennifer.hellier@kcl.ac.uk. The datasets analysed during the current study are available from GENDEP study team on reasonable request.

## Abbreviations

BMI: Body mass index
CI: Confidence interval
GENDEP: Genome Based Therapeutic Drugs for Depression
IG: Inverse Gaussian
IV: Instrumental variable
MADRS: Montgomery-Åsberg Depression Rating Scale
PH: Proportional hazards
RCT: Randomised controlled trial
SD: Standard deviation
SE: Standard error
TR: Threshold regression
TSRI: Two-stage residual inclusion
