# Supplementary material for: Estimating dose-response for time to remission with instrumental variable adjustment: the obscuring effects of drug titration in Genome Based Therapeutic Drugs for Depression Trial (GENDEP): clinical trial data
Source: Trials. 2020 Jan 3;21:10. doi: 10.1186/s13063-019-3810-9 (PMC6942263; doi:10.1186/s13063-019-3810-9)
Supplement: Supplementary file 1 — Additional file 1: Stata command routines. Table: GENDEP baseline demographic and clinical characteristics. [file 13063_2019_3810_MOESM1_ESM.docx]

**Supplementary Material: Appendices**

**(A) Stata command routines**

The IG model was fitted by maximum likelihood using a purpose written program in *Stata* 14. We used a numerical gradient in *Stata* called *lf* to find the maximum likelihood parameter estimates and their standard errors given in Results Table 3. The main routine is listed below. The main routine calls a subroutine that we call *ig2018*. The subroutine is listed below the main program. The terms *t* and *f* denote the censoring or failure time and failure indicator of the current observation, respectively. The terms *theta1* and *theta2* denote the two linear predictors representing distance and velocity, respectively. The model shown is adjusted for baseline depression MADRS score(dep_bl); duration of current depressive episode (dep_dur), sex(sex), age of depression onset (age_onset), body mass index (BMI) and averaged relative dose (dose). The survival function is estimated during the maximum likelihood routine as a temporary variable and can be extracted (s) post estimation

/* Subroutine */

capture program drop ig2018

program ig2018

version 14.0

args lnf theta1 theta2

tempvar A B E F G fig M N O lnS s

local y "$ML_y1"

local d "$ML_y2"

gen double `A' = ln((2*_pi)^-0.5)

gen double `B' = -1.5*ln(`y')

gen double `E' = ((exp(`theta1'))^2) / (`y')

gen double `F' = 2 * exp(`theta1')*(`theta2')

gen double `G' = (`y') * (`theta2')^2

gen double `fig' = (`theta1') + `A' + `B' - 0.5 * (`E' - `F' + `G')

gen double `M' = exp(`theta1')- (`y'*`theta2')

gen double `N' = -(exp(`theta1'))- (`y'*`theta2')

gen double `O' = (`y')^0.5

gen double `lnS' = ln(normprob(`M'/`O') - exp(`F')*normprob(`N'/`O'))

gen double `s' = exp(`lnS')

quietly replace `lnf' = (`d' * `fig') + ((1 - `d') * (`lnS'))

end

/* Main routine */

ml model lf ig2018 (theta1: t f = dep_bl dep_dur sex) (theta2: age_onset bmi dose)

ml max

**(B) Table: GENDEP baseline demographic and clinical characteristics**

Counts and percentages for categorical variables and mean and standard deviations for continuous variables. *higher MADRS score indicates more severe depression. MADRS: Montgomery-Åsberg Depression Rating Scale; BMI: Body Mass Index

|  |  | **Escitalopram** |  | **Nortriptyline** |  |
| --- | --- | --- | --- | --- | --- |
|  |  | n/mean | %/SD | n/mean | %/SD |
| **Number** |  | 196 |  | 184 |  |
| **Female** |  | 119 | 60.7% | 121 | 65.8% |
| **Age (years)** |  | 42.2 | 11.7 | 42.9 | 11.2 |
| **Married / cohabiting** |  | 113 | 57.7% | 105 | 57.1% |
| **Unemployed** |  | 44 | 22.5% | 38 | 21.6% |
| **Age of depression onset (years)** |  | 32.1 | 11.0 | 30.4 | 10.4 |
| **Number of depressive episodes** | 1 | 50 | 25.5% | 49 | 26.6% |
|  | 2 | 110 | 56.1% | 98 | 53.3% |
|  | 3 | 36 | 18.4% | 37 | 20.1% |
| **Duration of current episode (weeks)** |  | 21.2 | 18.7 | 19.5 | 16.1 |
| **History of taking any antidepressant** |  | 95 | 48.5% | 101 | 54.9% |
| **MADRS total score*** |  | 29.2 | 6.2 | 29.8 | 6.6 |
| **BMI (weight(kg)/height(m^2^))** |  | 25.4 | 4.6 | 25.8 | 5.6 |
